# Screening for adolescent idiopathic scoliosis: an information statement by the scoliosis research society international task force

**DOI:** 10.1186/1748-7161-8-17

**Published:** 2013-10-31

**Authors:** Hubert Labelle, Stephens B Richards, Marinus De Kleuver, Theodoros B Grivas, Keith D K Luk, Hee Kit Wong, John Thometz, Marie Beauséjour, Isabelle Turgeon, Daniel Y T Fong

**Affiliations:** 1 Orthopedic Division, Sainte-Justine University Hospital, University of Montreal, Montreal, Canada; 2 Department of Orthopaedic Surgery, Texas Scottish Rite Hospital for Children, Dallas, USA; 3 Department of Orthopaedic Surgery, University of Texas-Southwestern, Dallas, USA; 4 Department of Orthopedics, VU University Medical center, Amsterdam, the Netherlands and Sint Maartenskliniek, Nijmegen, Amsterdam, the Netherlands; 5 Orthopaedic and Trauma Department, Tzanio” General Hospital of Piraeus, Piraeus, Athens, Greece; 6 Department of Orthopaedics and Traumatology, The University of Hong Kong, Pokfulam, Hong Kong SAR, Hong-Kong, China; 7 Department of Orthopaedic Surgery, Yong Loo Lin School of Medicine, National University of Singapore, Singapore, Singapore; 8 Department of Orthopaedic Surgery, Children's Hospital of Wisconsin, Milwaukee, USA; 9 Research Center, Sainte-Justine University Hospital, University of Montreal, Montreal, Canada; 10 School of Nursing, The University of Hong Kong, Pokfulam, Hong Kong SAR, Hong-Kong, China

## Abstract

**Background:**

Routine screening of scoliosis is a controversial subject and screening efforts vary greatly around the world.

**Methods:**

Consensus was sought among an international group of experts (seven spine surgeons and one clinical epidemiologist) using a modified Delphi approach. The consensus achieved was based on careful analysis of a recent critical review of the literature on scoliosis screening, performed using a conceptual framework of analysis focusing on five main dimensions: technical, clinical, program, cost and treatment effectiveness.

**Findings:**

A consensus was obtained in all five dimensions of analysis, resulting in 10 statements and recommendations. In summary, there is scientific evidence to support the value of scoliosis screening with respect to technical efficacy, clinical, program and treatment effectiveness, but there insufficient evidence to make a statement with respect to cost effectiveness. Scoliosis screening should be aimed at identifying suspected cases of scoliosis that will be referred for diagnostic evaluation and confirmed, or ruled out, with a clinically significant scoliosis. The scoliometer is currently the best tool available for scoliosis screening and there is moderate evidence to recommend referral with values between 5 degrees and 7 degrees. There is moderate evidence that scoliosis screening allows for detection and referral of patients at an earlier stage of the clinical course, and there is low evidence suggesting that scoliosis patients detected by screening are less likely to need surgery than those who did not have screening. There is strong evidence to support treatment by bracing.

**Interpretation:**

This information statement by an expert panel supports scoliosis screening in 4 of the 5 domains studied, using a framework of analysis which includes all of the World Health Organisation criteria for a valid screening procedure.

## Introduction

Adolescent idiopathic scoliosis (AIS) is a complex 3-D deformation of the trunk, with a prevalence of 2-4%. Among patients with AIS, 8% to 9% will be treated by brace and 0.1% will need surgery using spinal instrumentation and fusion. Routine screening of scoliosis is a controversial subject and screening efforts vary greatly around the world [1], with mandatory scoliosis school screening programs (SSSP’s) in some areas, voluntary SSSP’s in others, while some countries recommend against. Currently, less than half of the states in the United States have legislated school screening, while national SSSP’s in Canada have been discontinued [2].

In 2008, the American Academy of Orthopaedic Surgeons (AAOS), the Scoliosis Research Society (SRS), the Pediatric Orthopaedic Society of North America (POSNA), and the American Academy of Pediatrics (AAP) issued an information statement [3] on screening in AIS, indicating that in 1996, the United States Preventive Services Task Force (USPSTF) concluded that there was insufficient evidence to make a recommendation for, or against, screening [4]. However, in 2004, the USPSTF changed their position and recommended against the routine screening of asymptomatic adolescents for idiopathic scoliosis [5]. The AAOS, SRS, POSNA, and AAP expressed concerns that this change in position by the USPSTF came in the absence of any significant change in the available literature, in the absence of any change in position statements by the AAOS, SRS, POSNA, and AAP, and in the absence of any significant input from specialists who commonly care for children with scoliosis. The AAOS, SRS, POSNA, and AAP did not support any formal recommendations against scoliosis screening, given the available literature.

In 2010, the SRS Presidential line determined that it would be worth exploring scoliosis screening from a multi-national perspective by creating an International Task Force. The goal of this article is to summarize the activities of the Task Force over the past 2 years, leading to the current consensus information statement on the value of scoliosis screening based on the available scientific literature.

## Material & methods

Seven SRS members were nominated by the Presidential line to join the Task Force (TF). Their selection was based on their known expertise on scoliosis screening: two members from USA, one from Canada, two from Europe, and two from Asia. In addition, two clinical epidemiologists were added to provide methodological support and one research assistant to coordinate the team’s effort. As one epidemiologist had clinical expertise on the subject, he was invited to join the TF for input, so that eight members voted on each issue discussed.

**Figure 1:**
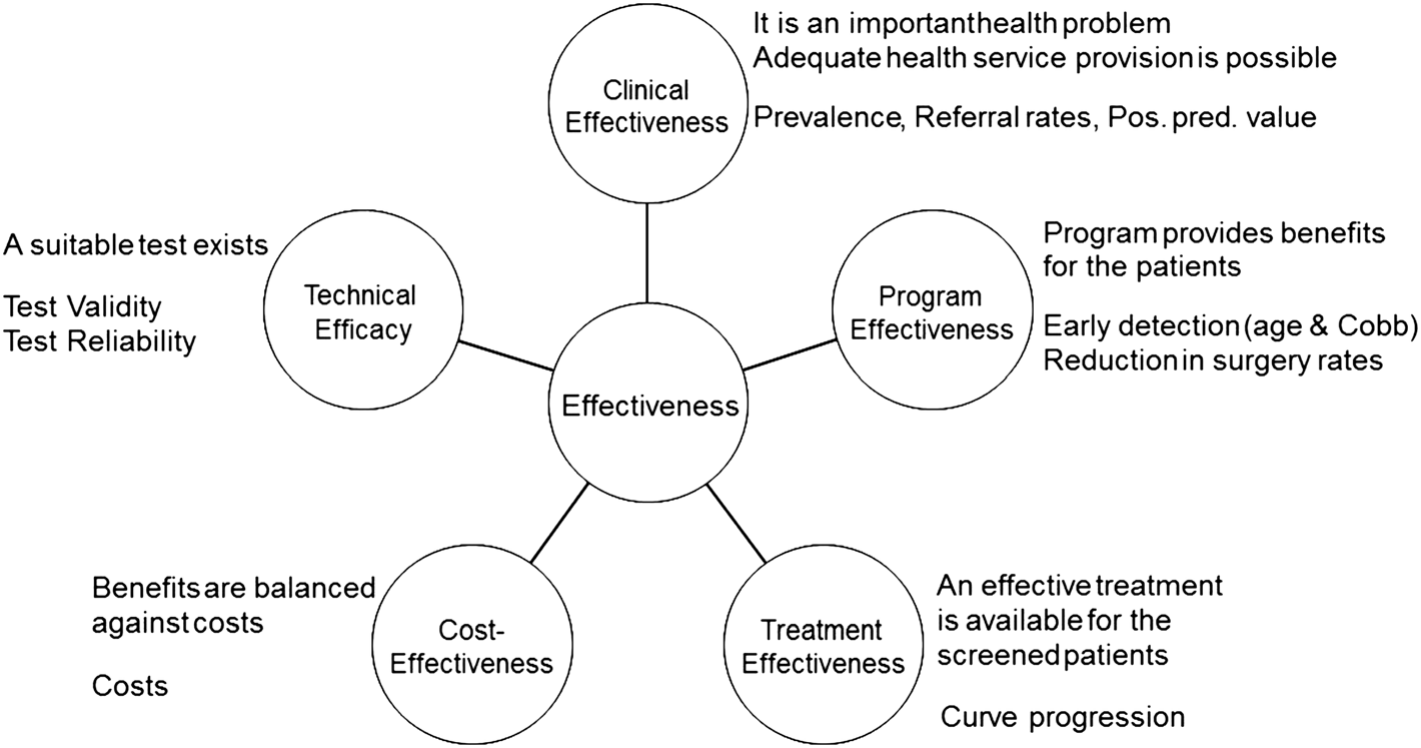
**The conceptual framework used to analyze scoliosis screening, focusing on *five *** **main dimensions: *Technical Efficacy*, which relates to the validity and reliability of the tests; *Clinical Effectiveness*, which describes the importance of the health problem and the consequences of screening on patient management and the health system; *Program Effectiveness *** **which refers to the benefits for the patients of adherence to the screening programs; *Cost-Effectiveness *** **where these benefits are balanced against costs for society and *Treatment Effectiveness *** **which concerns the benefits for patients of the available treatment modalities.**

Two conference calls were first held to establish the proper strategy to evaluate scoliosis screening. The first decision was to take advantage of a recent systematic review on the effectiveness of scoliosis screening [6], in which four databases were searched: Medline, Embase, CINAHL, EBM Reviews and Cochrane Central Registry of Controlled Trials. All relevant studies between 1950 and mid 2010 were selected and independently reviewed by two review authors (an epidemiologist and an orthopaedic surgeon). Key findings were summarized for each article in narrative format: study design, sample size, intervention (tools, personnel, setting, and population), main outcomes, key message, original author’s main conclusions, paper’s strengths and flaws, assessment of risk of bias, critical appraisal of the conclusions and clinical significance. Grading of the strength of evidence of individual studies was done using the Downs & Black 28-item tool [7]. The conceptual framework of analysis used in this systematic review was adopted by the Task Force, as it was considered ideal to analyze scoliosis screening, focusing on *five* main dimensions (Figure 1). This model has been used in other screening programs with success, and includes all of the World Health Organisation (WHO) criteria for a valid screening procedure [8].

Since treatment effectiveness was not included in the systematic review [6], and since a meta-analysis [9] providing a summary of the available evidence on brace treatment up to 1993 was available, a Medline database search of the French and English literature on brace treatment in AIS was performed from 1993 to 2012, to identify publications with level I, II or III evidence [10]. We therefore excluded all level IV (case series with no control group or with an historical control group) and all level V evidence (expert opinion). The search strategy copied the one used by Negrini et al [11], a Cochrane systematic review which analyzed 2 level I and II studies, one RCT and one prospective and controlled cohort.

Due to the wide geographical dispersion of the TF members, the second decision was to use an interactive website, Wikispaces, as the main tool to conduct the work. A secured website was thus set-up, to which only members were granted access. Instructions on how to navigate through the site were provided. In each page, members were able to browse the available information, download and upload files, and post comments directly on the page through a simple editing feature.

The online collaborative work began by answering two preliminary questions, the first on the goal for SSSP’s and the second on the analytical framework for screening program effectiveness (Figure 1). Each of the themes was sequentially opened for study by all members for a period of four to six weeks. The following information was extracted from the systematic review [6], and was made available for each on the website: a summary of evidence tables in PowerPoint format, each article studied in PDF format and the detailed critical evaluation of each article including the Downs & Black 28-item score. For each theme, members were invited to answer a series of statements derived from the critical review of the literature, with an agreement scale (Strongly agree, agree, disagree, strongly disagree), and designed to guide members through the literature review and make their own expert opinion based on the literature review. Each member was asked to individually respond to each statement, and return his answers confidentially to the website manager by e-mail. For each section, once all responses were returned and compiled, the results were presented on the website. A period of four weeks was then allowed in each section for members to freely discuss the results and expand on the areas of disagreement. Following this discussion, a wrap-up on the areas of consensus and disagreement was done by the lead author to conclude each website section. In addition, four face-to-face meetings of the TF were held in 2011 and 2012, allowing members to get training on use of the website, freely discuss areas of controversy revealed on the website and prepare the current consensus information statement.

## Results

Additional file 1: Table S1 is a summary of each set of statements for each of the five dimensions studied, with agreement results in each dimension. For the preliminary questions, all agreed that Scoliosis screening should be aimed at identifying suspected cases of scoliosis that will be referred for diagnostic evaluation and confirmed, or ruled out, with a clinically significant scoliosis (>10° Cobb angle [12]). Participants also agreed that conclusions on screening programs effectiveness should be provided by carefully examining the available literature according to the conceptual framework. *For technical efficacy*, 42 articles were reviewed (Additional file 2, A1-A42) and five questions asked. *For clinical effectiveness*, 58 articles were reviewed (Additional file 2, A43-A98) and 12 questions asked. This dimension is defined as the extent to which an intervention does what it is aimed to do, in the clinical setting [13]. *For program effectiveness*, 11 comparative studies between participants and non participants to screening programs were reviewed (Additional file 2, A99-A109). Seven questions were asked for two separate outcomes. In outcome A, the characteristics of the patients at time of detection/diagnostic (in terms of age and Cobb angle) were compared in screened and unscreened samples of participants to determine if the goal of “early detection” is achieved by these screening programs. In outcome B, the reduction in the number of required surgeries was assessed, which is the “ultimate goal” of screening for scoliosis [14]. *For cost effectiveness*, only 8 articles were available for review (Additional file 2, A110-A117) and seven questions were asked. Finally, *for treatment effectiveness*, our literature search revealed 24 articles (Additional file 2, A118-A141) on brace treatment in AIS with level I, II or III evidence [10], leading to 7 questions.

## Discussion

This critical analysis of the current literature revealed many areas of agreement in all five dimensions which will be further discussed.

### Technical efficacy

Members agreed on the tools/techniques to be included in the review and on the scoliometer as the best tool (used alone with an Adams Forward bending test) in terms of reliability and validity to measure trunkal asymmetry (as proxy for spinal deformities). There is evidence that scoliometer measures in the sitting position may be useful, especially in patients presenting with important leg length discrepancy. Members agreed on the relevant reliability and validity values to evaluate the tools and techniques of trunkal asymmetry measures (as a proxy for spinal deformities). Work by Lee et al [15], (which is the first study providing graphs of sensitivity and PPV according to the threshold for positive cases) does not invalidate the general recommendation about the use of the scoliometer. It was noted that there is controversial evidence that using Moiré Topography in combination with the scoliometer may improve the sensitivity of the screening protocol. TF members agreed that the recommended threshold for the use of the scoliometer should be between 5° and 7° when used alone. The issue of the recommended age for screening needs further clarification. The literature is difficult to interpret but members agreed that screening should be conducted two years before onset of menses. Members also agreed on the availability of low to moderate quality evidence to support measurement estimates. Threshold determination for positive cases resulted in an interval of values and the lack of prospective studies was noted.

### Clinical effectiveness

Members agreed on the definition of Clinical Effectiveness, with the limitation that SSSP’s discover children with trunk asymmetry, of which a portion will have scoliosis. This discordance between spinal and surface asymmetry/deformity in younger children leads to over-referrals from SSSP’s and is the main cause of the ongoing controversy over its application. Therefore, future studies of screening programs should try to determine how many children with trunk asymmetry have scoliosis. There was also agreement on the retained measures (prevalence, referral rate, positive predictive value) for this dimension, but one member thought that Sensitivity should be added as another measures for clinical effectiveness. For question 3, there was consensus on the definition for prevalence of scoliosis, but one member disagreed since usually no treatment is offered for curves <25°, and one member thought that the percentage of curves >10° among those screened may over-estimate the true prevalence. On question four, a majority of members disagreed with the proposed definition of referral rates in orthopedics/specialized clinic, since patients who are referred and those who are actually seen represent two distinctly separate situations. As the direct output of screening programs, referral rate should be defined as the percentage of screened individuals with a positive test. For question 5 there was consensus on the proposed definition for positive predictive value, but one member questioned whether this value should be calculated for diagnosed cases of scoliosis > 10°, or for cases that need clinical follow-up or treatment (for example, >20°). To ease comparison, there was consensus that there are advantages to rely on the standard definition of scoliosis.

There was consensus that Clinical Effectiveness is only one of the dimensions of Effectiveness and that any decision on the global value of screening programs should be taken by careful examination of all five dimensions. For question seven, looking at the strongest evidence on the studies providing estimates of clinical effectiveness measures, members supported that the large retrospective follow-up study from Luk et al [16]. constitutes very strong evidence that should lead decisions related to clinical effectiveness of screening. Fong et al’s very rigorous meta-analysis [17] provides estimates from 36 lower quality studies and illustrates the heterogeneity of the available studies. Age, gender, screening tests used, study size, publication date, screening setting and even latitude may be factors to investigate. Nevertheless, in these studies [16,17] as well as in 23/36 studies included in Fong’s meta-analysis, the authors considered these as «adequate values» and concluded on the Clinical Effectiveness of SSSP’s.

According to WHO’s [8] criteria, the condition should be an important health problem, both in terms of morbidity and disease frequency. Therefore, prevalence is one important aspect of the decision. In addition, facilities for diagnosis and treatment should be available, or by extension, the volume and characteristics of patients referred in orthopaedics for evaluation and diagnosis should be considered as “appropriate” or manageable by the health care system in order to provide adequate care. As for question 11, there was consensus that scoliosis is a condition amenable to screening, but members recognize that this is an expert opinion from spine caregivers is based on low to moderate evidence and that perspectives could be different if respondents were independent non-spine caregivers critically analyzing the same evidence.

### Program effectiveness

Members noted the paucity of comparative studies in the field of scoliosis screening. Based on the available literature, they agreed that screen detected scoliosis patients are younger and less severely affected at time of detection and diagnosis than otherwise detected patients. However, even if the reported results are highly consistent, members were not unanimous in grading the proposed level of evidence as moderate, since individual studies were low quality. TF members also agreed that reduction in the number of surgeries is a major goal of screening programs. There are no strong studies assessing this outcome, because of poor design and heterogeneity in results. All agreed that because this question could have considerable impact for the patient and health system, they should therefore put forth a conclusive statement on the issue. TF members did not unanimously agree on the proposed statement of the likelihood of an association between screening programs and reduction in surgery rate, thus a consensus on the following reformulated recommendation: there is some evidence, although low and questionable that screen detected scoliosis patients are less likely to be recommended for surgery than otherwise detected patients.

### Cost effectiveness

Members recognized that few studies focused on cost analysis as their main objectives. Available studies need to be analyzed carefully since the reported “total costs” are based on different perspectives, making direct comparison of results difficult: program costs, program + diagnostic costs, program + diagnostic + follow-up costs, program + diagnostic + follow-up + treatment costs. To ease comparison, members were provided with cost-charts adjusted for mean inflation and converted to 2010 USD [18]. Looking only at program costs, i.e. direct costs of the screening intervention, costs ranged from 50 cents to $12/per screened for FBT only or the scoliometer, $20 for the protocol including Moiré topography. In absolute terms, these costs were considered reasonably low. However, costs increase substantially when considered for each child brought to treatment. These numbers ought to be analyzed in correspondence with measures of effectiveness, i.e. with an evaluation of decreased overall expense because of avoided surgeries and major interventions. There were limited data exploring this question, with two studies reviewed on cost-effectiveness, which actually support cost-effectiveness of screening, but based on disputable assumptions on bracing effectiveness. Members supported further investigation by comparing two comparable settings, one with screening programs and one without (comparable cohorts from comparable health and economic systems) or by comparing those screened with those who had defaulted screening or who had sought alternative non-operative treatment, in the same health service area. The SRS could be a scientific and operation leader in such an endeavor.

### Treatment effectiveness

Finally, with respect to the controversial theme of brace treatment efficacy (Additional file 2, A118-A141), the Task Force consensus indicates that based on level II and III evidence, there is scientific literature supporting the short and long term efficacy of full-time brace wear to prevent progression of AIS. With the recently published results of the BrAIST multicenter NIH trial [19], there is now level I evidence to support the efficacy of brace treatment in AIS.

## Conclusions

After a critical review of the available evidence, the SRS International Task Force on Scoliosis screening, supported by the SRS Board of Directors, makes the following statements and recommendations regarding scoliosis screening:

1- Scoliosis screening is recommended as valuable in the following domains: technical efficacy, clinical, program and treatment effectiveness. The existing literature does not provide sufficient evidence to make a statement with respect to cost effectiveness.

2- Scoliosis screening should be aimed at identifying suspected cases of scoliosis (labeled as “positive cases” according to clearly defined criteria) that will be referred for diagnostic evaluation and confirmed, or ruled out, with a clinically significant scoliosis (>10 degrees of Cobb angle). Females should be screened twice, at age 10 and 12, and boys once, at age 13 or 14 [3].

3- The scoliometer is currently the best tool available for scoliosis screening.

4- There is moderate evidence to recommend referral with scoliometer values between 5° and 7°, or greater. The addition of Moire topography may improve sensitivity.

5- There is moderate evidence that the use of scoliosis screening allows for detection and referral of patients with AIS at an earlier stage of the clinical course, in terms of younger age and/or lower Cobb angle.

6- There is evidence that scoliosis patients detected by screening are less likely to need surgery than those patients who did not have screening.

7- Prevalence, Referral rates and Positive Predictive Value of current screening tools in screened children reach adequate values (expert opinion), so as to consider scoliosis a condition suitable for screening.

8- There is strong evidence to support the value of bracing for the treatment of AIS.

9- Continued work to determine minimum standards and targets (in terms of referral rates and Positive Predictive Value) is needed for screening programs.

10- Further investigation on cost-effectiveness of screening programs should be obtained by studying comparable settings: one with scoliosis screening, and one without.

## Competing interests

The authors of this article have no financial or personal relationship with other people or organizations that could inappropriately influence (bias) their work, except for Hubert Labelle who has the following financial relationship to disclose: Stock ownership with Spinologics Inc.

## Authors’ contributions

HL initiated the idea for a review article on scoliosis screening and drafted the article. All other co-authors contributed their professional skills, reviewed the article and partially contributed in drafting of the manuscript adding certain references. All authors have read and approved the final manuscript.

## Supplementary Material

Additional file 1: Table S1 Click here for file

Additional file 2 Annex. Click here for file
