# Supplementary material for: Screening for adolescent idiopathic scoliosis: an information statement by the scoliosis research society international task force
Source: Scoliosis. 2013 Oct 31;8:17. doi: 10.1186/1748-7161-8-17 (PMC3835138; doi:10.1186/1748-7161-8-17)
Supplement: Additional file 1: Table S1 [file 1748-7161-8-17-S1.docx]

**Additional file 1: Table S1**

| **Technical Efficacy:** | **Strongly agree** | **Agree** | **Disagree** | **Strongly Disagree** | **Abstain** |
| --- | --- | --- | --- | --- | --- |
| **QUESTION 1:**  After reviewing the literature do you agree that only the Adams Foward Bending Test (AFBT), the Scoliometer and Moiré Topography should be evaluated, since there is insufficient data for the other techniques? | **87·5%** | **12·5%** | **0%** | **0%** | **0%** |
| **QUESTION 2:** Do you agree that analysis of the efficacy of these technique/tool(s) should be based on reliability and validity measures? | **75%** | **12·5%** | **0%** | **0%** | **12·5%** |
| **QUESTION 3**: Do you agree that the best currently available technique/tool for scoliosis screening is the Scoliometer used on a Forward Bending Test? | **37.5%** | **37·5%** | **12·5%** | **12·5%** | **0%** |
| **QUESTION 4:** Do you agree that the threshold for positive cases using a Scoliometer should be between 5 and 7 degrees? | **37·5%** | **37·5%** | **12·5%** | **0%** | **12·5%** |
| **QUESTION 5:** Looking at the best evidence available, do you agree that there are sufficiently precise estimates of reliability and sensitivity measures coming from low to moderate quality studies? | **25%** | **62·5%** | **0%** | **0%** | **12·5%** |

| **Clinical Effectiveness:**  **Definition: Clinical Effectiveness is defined as the extent to which an intervention does what it is aimed to do, in the clinical setting. Therefore, Clinical Effectiveness of scoliosis screening programs could be defined as an appreciation on how good are the programs in achieving the goal of identifying patients with scoliosis among the individuals submitted to the screening intervention.** | **Strongly agree** | **Agree** | **Disagree** | **Strongly Disagree** | **Abstain** |
| --- | --- | --- | --- | --- | --- |
| **Question 1:** Do you agree with this definition of Clinical Effectiveness of scoliosis screening? | **25%** | **75%** | **0%** | **0%** | **0%** |
| **Question 2:** Do you agree that Clinical Effectiveness is a dimension usually operationalized in the scoliosis screening literature by three measures: prevalence, referral rates and positive predictive value in the screened population? | **50%** | **50%** | **0%** | **0%** | **0%** |
| **Question 3:** Do you agree that prevalence of scoliosis should be defined as: “The finding of an idiopathic scoliosis curve of more than 10^o^ Cobb angle, as measured on a postero-anterior standing radiograph (Kane’s criteria)^12^ amongst the total children screened”? | **37·5%** | **50%** | **12·5%** | **0%** | **0%** |
| **Question 4:** Since traditionally, positives cases identified from scoliosis screening programs are referred in orthopedics for evaluation, do you agree that referral rates in orthopedics/specialized clinic should be defined as: “The percentage of children referred and actually seen in orthopedics after a positive screening test”? | **25%** | **12·5%** | **62·5%** | **0%** | **0%** |
| **Question 5:** Since Positive predictive value is classically defined as the probability of actually having the disease if tested positive on screening, do you agree that, in the context of scoliosis screening programs, positive predictive value should be defined as: “the percentage of diagnosed scoliosis according to Kane’s criteria^12^ in positively tested children from the screened population."? | **50%** | **37·5%** | **12·5%** | **0%** | **0%** |

| **Clinical Effectiveness: (*continued)***  **In the literature on scoliosis screening, Clinical Effectiveness is the main, and often the only outcome considered in support of the value of the screening programs.** | **Strongly agree** | **Agree** | **Disagree** | **Strongly Disagree** | **Abstain** |
| --- | --- | --- | --- | --- | --- |
| **Question 6:** Do you agree that Clinical Effectiveness is only *one* of the dimensions of the Effectiveness construct and that any decision on the global value of screening programs should be taken by careful examination of all 5 dimensions listed in Figure1? | **37·5%** | **62·5%** | **0%** | **0%** | **0%** |
| **Question 7:** Do you agree the meta-analysis by Fong et al. 2010^17^ provides the strongest available evidence for the estimation of the three main measures of clinical effectiveness: *prevalence, referral rates and positive predictive value in the screened patients*? | **12·5%** | **62·5%** | **12·5%** | **0%** | **12·5%** |
| **Question 8:** Do you agree that the available literature provides satisfactory estimates of prevalence, referral rates and positive predictive value in screened children? | **12·5%** | **50%** | **37.5%** | **0%** | **0%** |
| **Question 9:** Do you agree that designing and conducting other prevalence studies are not likely to change the conclusions on this topic? | **12·5%** | **37·5%** | **37·5%** | **0%** | **12·5%** |
| **Question 10:** Do you agree that researchers should further investigate the underlying reasons for the observed heterogeneity in the data? | **50%** | **25%** | **25%** | **0%** | **0%** |
| **Question 11:** Do you agree, that Prevalence, Referral rates and Positive Predictive Value of current screening tools in screened children reach adequate values (expert opinion), so as to consider scoliosis a condition suitable for screening? | **25%** | **50%** | **12·5%** | **0%** | **12·5%** |
| **Question 12:** Do you agree that minimum standards and targets in terms of referral rates and Positive Predictive Value should be determined for screening programs in order to choose the best tools and procedures for screening and guide screening policies? | **50%** | **50%** | **0%** | **0%** | **0%** |

| **Program Effectiveness:**  **Outcome A:** **Characteristics of the patients at time of detection/diagnostic** | **Strongly agree** | **Agree** | **Disagree** | **Strongly Disagree** | **Abstain** |
| --- | --- | --- | --- | --- | --- |
| **QUESTION 1:** The first question under this topic should be: “Are screen detected scoliosis patients younger and less severely affected at time of detection and diagnosis than otherwise detected patients?” | **87·5%** | **12·5%** | **0%** | **0%** | **0%** |
| **QUESTION 2:** Do you agree that according to the available literature, the use of scoliosis screening allows to detect and refer patients with AIS at an earlier stage of the clinical course (than otherwise detected), in terms of younger age and/or lower Cobb angle? | **75%** | **25%** | **0%** | **0%** | **0%** |
| **QUESTION 3:** Early detection is an important patient outcome because it allows early intervention, whatever the treatment decision is: brace or other (physiotherapy, exercise, etc) or even observation? | **87·5%** | **12·5%** | **0%** | **0%** | **0%** |
| **QUESTION 4:** To qualify the underlying evidence for this question as moderate since results are consistent among low to moderate quality studies? | **25%** | **62·5%** | **12·5%** | **0%** | **0%** |
| **Outcome B: Reduction in the number of required surgeries** |  |  |  |  |  |
| **QUESTION 5:** The second question under this topic should be: Are screen detected scoliosis patients less likely to be recommended for surgery than otherwise detected patients? | **50%** | **50%** | **0%** | **0%** | **0%** |
| **QUESTION 6:** According to the available data, the use of scoliosis screening seems associated with a decrease in the number of patients requiring surgery for AIS? | **25%** | **62·5%** | **12·5%** | **0%** | **0%** |
| **QUESTION 7:** Although the evidence is low (poor study design study, heterogeneity in results), a conclusion/recommendation should be formulated by the Task Force on this question considering its considerable impact on the patients and the health system? | **37·5%** | **62·5%** | **0%** | **0%** | **0%** |

| **Cost Effectiveness**: 8 articles published between 1997-2010 present cost evaluation as one of their main objective^annexA118–A141^. Others may have published ad hoc data. Only two have formulated hypotheses in regards to cost-effectiveness, based on assumptions regarding the effectiveness of screening and bracing in order to avoid surgery. | **Strongly agree** | **Agree** | **Disagree** | **Strongly Disagree** | **Abstain** |
| --- | --- | --- | --- | --- | --- |
| **Question 1:** Do you agree on the choice of included studies? | **25%** | **75%** | **0%** | **0%** | **0%** |
| **Question 2:** Do you agree that, based on the available literature, it is difficult to globally conclude on cost-effectiveness of scoliosis screening? | **75%** | **25%** | **0%** | **0%** | **0%** |
| **Question 3:** Do you agree that these assumptions are disputable? | **62·5%** | **37·5%** | **0%** | **0%** | **0%** |
| **Question 4:** Do you agree that decision about cost-effectiveness is relevant in a particular economic and political context? | **50%** | **50%** | **0%** | **0%** | **0%** |
| **Question 5:** Do you agree that having access to a detailed description of involved costs is however useful to identify elements of the intervention that are the most cost-intensive and that consume the most resources? | **37·5%** | **62·5%** | **0%** | **0%** | **0%** |
| **Question 6:** Do you agree that further investigation on cost-effectiveness of screening programs should be obtained by comparing two comparable settings (comparable cohorts from comparable health and economic systems): one with scoliosis screening and one without, on a specified list of cost items to be included, involving prospective cohorts of patients followed-up to maturity for outcomes characterization? | **50%** | **37·5%** | **12·5%** | **0%** | **0%** |
| **Question 7:** Do you agree that the SRS should support such a research project? Scientifically? Operationally? Financially? | **50%** | **50%** | **0%** | **0%** | **0%** |

| **Treatment Effectiveness**: 24 articles level I II and III on brace were selected and reviewed. To simplify the process without diverging too much from the original systematic review protocol, we have implemented the following changes:   - We considered that Rowe’s meta-analysis provides a good summary of the available evidence up to 1993. We suggest that you read again this study (Rowe et al. JBJS 1997)^9^ in addition to the literature review that will follow. - We therefore searched the Medline database only from 1993 to 2012. - We used a similar but more inclusive search strategy than Negrini et al. in their 2010 Cochrane review^11^. - We selected only comparative studies with control group(s), discarding case series, since judging effectiveness requires a comparison. | **Strongly agree** | **Agree** | **Disagree** | **Strongly Disagree** | **Abstain** |
| --- | --- | --- | --- | --- | --- |
| **Question 1:** **Do you agree with these methodological choices for the study selection with regards to brace treatment effectiveness?** | **50%** | **12·5%** | **37·5%** | **0%** | **0%** |
| **Question 2:** **Do you think that some important studies were forgotten in the selection process? Please specify.** | **12·5%** | **37·5%** | **50%** | **0%** | **0%** |
| **Question 3: All included studies quantified treatment success using clearly defined outcome measures. However, since several valuable publications pre-dated the publication of the SRS bracing criteria^19^, we did not confine our selection to these criteria. Do you agree?** | **25%** | **75%** | **0%** | **0%** | **0%** |
| **Question 4: In addition to Nachemson et al. 1995 study and Rowe’s meta-analysis in 1997, a few studies concluded on the post-treatment clinical outcomes. Do you believe that these properly support the efficacy of brace treatment?** | **25%** | **37·5%** | **37·5%** | **0%** | **0%** |
| **Question 5:** **Some studies investigated the long-term effect of brace treatment. Do you believe that these provide further support in favor of brace effectiveness?** | **12·5%** | **87·5%** | **0%** | **0%** | **0%** |
| **Question 6: Five studies compared part-time with full-time bracing and one proposed a dose-response relationship. Do you believe that these studies add convincing evidence in favor of full-time brace prescription?** | **50%** | **25%** | **25%** | **0%** | **0%** |
| **Question 7: Was this process sufficiently thorough to fulfill the SRS mandate? Please provide suggestions if applicable.** | **25%** | **62·5%** | **12·5%** | **0%** | **0%** |
