# Supplementary material for: Screening for adolescent idiopathic scoliosis: an information statement by the scoliosis research society international task force
Source: Scoliosis. 2013 Oct 31;8:17. doi: 10.1186/1748-7161-8-17 (PMC3835138; doi:10.1186/1748-7161-8-17)
Supplement: Additional file 2 — Annex. [file 1748-7161-8-17-S2.docx]

**Annex**

A1 Adair IV, van Wijk MC, Armstrong WD. Moiré topography in scoliosis screening. *Clin Ortho* 1978; **129**: 165–171.

A2 Amendt LE, Ause-Ellias KL, Eybers JL, Wadsworth CT, Nielsen DH, Weinstein SL. Validity and reliability testing of the Scoliometer. *PhyTher* 1990; **70**: 108–17.

A3 Bunnell WP. An objective criterion for scoliosis screening. *J Bone Joint Surg Am* 1984; **66**: 1381.

A4 Bunnell WP. Outcome of spinal screening. *Spine* 1993; **18**: 1572–80.

A5 Burwell RG, James NJ, Johnson F, Webb JK, Wilson YG. Standardized trunk asymmetry scores. A study of back contour in healthy school children*. J Bone Joint Surg Br* 1983; **65**: 452–63.

A6 Burwell RG, Aujla RK, Kirby AS, Moulton A, Webb JK. The early detection of adolescent idiopathic scoliosis in three positions using the scoliometer and real-time ultrasound: should the prone position also be used? *Stud Health Technol Inform* 2002; **88**: 74–80.

A7 Connolly BH, Michael BT. Early detection of scoliosis. A neurological approach using the asymmetrical tonic neck reflex. *PhyTher* 1984; **64**: 304–7.

A8 Cooke ED, Carter LM, Pilcher MF. Identifying scoliosis in the adolescent with thermography: a preliminary study. *Clin Orthop Relat Res* 1980; **148:** 172–6.

A9 Cote P, Kreitz BG, Cassidy JD, Dzus AK, Martel J. A study of the diagnostic accuracy and reliability of the Scoliometer and Adam's forward bend test. *Spine* 1988; **23**: 796–802; discussion 803.

A10 Daler S, Huber F. Topographie Moiré et déviations vertébrales (étude en milieu scolaire). *Ann Readapt Med Phys* 1984; **26**: 319–26.

A11 De Wilde L, Plasschaert F, Cattoir H, Uyttendaele D. Examination of the back using the Bunnell scoliometer in a Belgian school population around puberty. *Acta Orthop Belg* 1998; **64**: 136–43.

A12 El-Sayyad MM. Comparison of roentgenography and moire topography for quantifying spinal curvature. *Phy Ther* 1986; **66**: 1078–82.

A13 Flynn JC, Riddick MF, Price CT, Keller TL. Present status of scoliosis screening in Florida schools. *J Fla Med Assoc* 1985; **72**: 847–51.

A14 Grivas TB, Vasiliadis ES, Polyzois VD, Mouzakis V. Trunk asymmetry and handedness in 8245 school children. *Pediatr Rehabil* 2006; **9**: 259–66.

A15 Grivas TB, Vasiliadis ES, Mihasc Maziotou C, Triandafyllopoulos G. Back trunk morphology in 3301 children aged 3-9 years old. *Stud Health Technol Inform* 2008; **140**: 29–32.

A16 Grossman TW, Mazur JM, Cummings RJ. An evaluation of the Adams forward bend test and the scoliometer in a scoliosis school screening setting. *J Pediatr Orthop* 1995; **15**: 535–8.

A17 Huang SC, Chen PQ, Yu KS, Liu TK. Effectiveness of scoliometer in school screening for scoliosis. *J Formos Med Assoc* 1988; **87**: 955–9.

A18 Huang SC. Cut-off point of the Scoliometer in school scoliosis screening. *Spine* 1997; **22**: 1985–9.

A19 Karachalios T, Sofianos J, Roidis N, Sapkas G, Korres D, Nikolopoulos K. Ten-year follow-up evaluation of a school screening program for scoliosis. Is the forward-bending test an accurate diagnostic criterion for the screening of scoliosis? *Spine* 1999; **24**: 2318–24.

A20 Korovessis PG, Stamatakis MV. Prediction of scoliotic cobb angle with the use of the scoliometer. *Spine* 1996; **21**: 1661–6.

A21 Lee CF, Fong DY, Cheung KM et al. Referral criteria for school scoliosis screening: assessment and recommendations based on a large longitudinally followed cohort. *Spine* 2001; **35**: E1492–8.

A22 Murrell GA, Coonrad RW, Moorman CT 3^rd^, Fitch RD. An assessment of the reliability of the Scoliometer. *Spine* 1993; **18**: 709–12.

A23 Nissinen M, Heliovaara M, Ylikoski M, Poussa M. Trunk asymmetry and screening for scoliosis: a longitudinal cohort study of pubertal schoolchildren. *Acta Paediatr* 1993; **82**: 77–82.

A24 Nissinen M, Heliovaara M, Seitsamo J, Alaranta H, Poussa M. Anthropometric measurements and the incidence of low back pain in a cohort of pubertal children. *Spine* 1994; **19**: 1367–70.

A25 Orderberg G, Hansson G. The scoliometer-experiences of interobserver reliability and correlation with radiographic curve in idiopathic scoliosis. *Acta Orthop Scand* 1991; **62**(suppl 246): 21.

A26 Pearsall DJ, Reid JG, Hedden DM. Comparison of three noninvasive methods for measuring scoliosis. *Phy Ther* 1992; **72**: 648–57.

A27 Pruijs JE, Keessen W, van der Meer R, van Wieringen JC, Hageman MA. School screening for scoliosis: methodologic considerations. Part 1: External measurements. *Spine* 1992; **17**: 431–6.

A28 Pruijs JE, Hageman MA, Keessen W, Van der Meer R, Van Wieringen JC. Spinal rotation meter: development and comparison of a new device. *Acta Orthop Belg* 1995; **61**: 107-12.

A29 Pruijs JE, Keessen W, van der Meer R, van Wieringen JC. School screening for scoliosis: the value of quantitative measurement. *Eur Spine J* 1995; **4**: 226–30.

A30 Ruggerone M, Austin JH. Moire topography in scoliosis. Correlations with vertebral lateral curvature as determined by radiography. *Phy Ther* 1986; **66**: 1072–7.

A31 Sahlstrand T. The clinical value of Moire topography in the management of scoliosis. *Spine* 1986; **11**: 409–17.

A32 Scutt ND, Dangerfield PH, Dorgan JC. The relationship between surface and radiological deformity in adolescent idiopathic scoliosis: effect of change in body position. *Eur Spine J* 1996; **5**: 85–90.

A33 Shinoto A. Quantitative analysis of scoliotic deformity by Moire method. *Nippon Seikeigeka Gakkai Zasshi. J Orthop Sci* 1981; **55**: 1703–18.

A34 Stokes IA, Moreland MS. Measurement of the shape of the surface of the back in patients with scoliosis. The standing and forward-bending positions. *J Bone Joint Surg Am*  1987; **69**: 203–11.

A35 Stokes IA, Moreland MS. Concordance of back surface asymmetry and spine shape in idiopathic scoliosis. *Spine* 1989; **14**: 73–8.

A36 Thompson F, Walsh M & Colville J. Moire topography: a method of screening for adolescent idiopathic scoliosis. *Ir Med J* 1985; **78**: 162–5.

A37 Upadhyay SS, Burwell RG, Webb JK. Hump changes on forward flexion of the lumbar spine in patients with idiopathic scoliosis. A study using ISIS and the Scoliometer in two standard positions. *Spine* 1988; **13**: 146–51.

A38 Viviani GR, Budgell L, Dok C, Tugwell P. Assessment of accuracy of the school screening examination. *Am J Public Health* 1985; **74**: 497–498.

A39 Willner S. Moire topography for the diagnosis and documentation of scoliosis. *Acta Orthop Scand* 1979; **50**: 295–302.

A40 Willner S. Moire topography--a method for school screening of scoliosis. *Arch Orthop Trauma Surg* 1979; **95**: 181–5.

A41 Willner S. A comparative study of the efficiency of different types of school screening for scoliosis. *Acta Orthop Scand* 1982; **53**: 769–74.

A42 Wong HK, Balasubramaniam P, Rajan U, Chng SY. Direct spinal curvature digitization in scoliosis screening--a comparative study with Moire contourgraphy. *J Spinal Disord* 1997; **10**: 185–92.

**Clinical Effectiveness:**

A43 Abbott EV. Screening for scoliosis: a worthwhile preventive measure. Canadian Journal of Public Health. *Revue Canadienne de Sante Publique* 1977; **68** : 22–5.

A44 Abo-Bakr A, Al-Mazyiad A, Al-Hussein M, Al-Sudairy R, Krimly M, Pravichandra JP. Adolescent idiopathic scoliosis screening of schoolgirls. *Ann Saudi Med* 1992; **12**: 555–7.

A45 Adler NS, Csongradi J, Bleck EE. School screening for scoliosis. One experience in California using clinical examination and moire photography. *West J Med* 1984; **141**: 631–3.

A46 Ascani E, Salsano V, Giglio G. The incidence and early detection of spinal deformities. A study based on the screening of 16,104 schoolchildren. *Ital J Orthop Traumatol*  1977; **3**: 111–7.

A47 Asher M, Green P, Orrick J. A six-year report: spinal deformity screening in Kansas school children. *J Kans Med Soc* 1980; **81**: 568–71.

A48 Bremberg S, Nilsson-Berggren B. School screening for adolescent idiopathic scoliosis. *J Pediatr Ortho.* 1986; **6**: 564–7.

A49 Chan A, Moller J, Vimpani G, Paterson D, Southwood R, Sutherland A. The case for scoliosis screening in Australian adolescents. *Med J Aust* 1986; **145**: 379–83.

A50 Corea JR. Should we screen for scoliosis in Sri Lanka? *Ceylon Med J* 1991; **36**: 68.

A51 Dickson RA, Stamper P, Sharp AM, Harker P. School screening for scoliosis: cohort study of clinical course.  *BMJ* 1980; **281**: 265–7.

A52 Dickson RA. Scoliosis in the community. *Br Med J (Clin Res Ed)* 1983; **286**: 615–8.

A53 Drennan JC, Campbell JB, Ridge H. Denver: a metropolitan public school scoliosis survey. *Pediatrics* 1977; **60**: 193–6.

A54 Dunn BH, Hakala MW, McGee ME. Scoliosis screening. *Pediatrics* 1978; **61**: 794–7.

A55 Francis RS, Bryce GR. Screening for musculoskeletal deviations--a challenge for the physical therapist. The Utah Study. *Phys Ther* 1987; **67**: 1221–5.

A56 Francis RS. Scoliosis screening of 3,000 college-aged women. The Utah Study--phase 2. *Phys Ther* 1988; **68**: 1513–6.

A57 Frost J, Shiratori H, Lam J. Scoliosis screening pilot project-A. Preliminary report to development of a statewide school program. *Hawaii Med J* 1978; **37**: 361–2.

A58 Goldberg C, Thompson F, Dowling F, Regan BF, Blake NS. Pilot study for a scoliosis screening project in South Dublin*. Ir Med J* 1980; **73**: 265–8.

A59 Goldberg CJ, Dowling FE, Fogarty EE, Moore DP. School scoliosis screening and the United States Preventive Services Task Force. An examination of long-term results. *Spine* 1995; **20**: 1368–74.

A60 Gore DR, Passehl R, Sepic S, Dalton A. Scoliosis screening: results of a community project. *Pediatrics* 1981; **67**: 196–200.

A61 Green PB. Spine deformity screening in Kansas. *J Sch Health* 1979; **49**: 56–7.

A62 Grivas TB, Koukos K, Koukou UI, Maziotou C, Polyzois BD. The incidence of idiopathic scoliosis in Greece--analyais of domestic school screening programs. *Stud Health Technol Inform* 2002; **91**: 71–5.

A63 Grivas TB, Samelis P, Polyzois BD, Giourelis B, Polyzois D. School screening in the heavily industrialized area--Is there any role of industrial environmental factors in idiopathic scoliosis prevalence? *Stud Health Technol Inform* 2002; **91**: 76–80.

A64 Gurr JF. A school screening program that works. *Can Nurse* 1977; **73**: 24–9.

A65 Hazebroek-Kampschreur AA, Hofman A, van Dijk AP, van Linge B. Prevalence of trunk abnormalities in eleven-year-old schoolchildren in Rotterdam, The Netherlands. J *Pediatr Orthop*  1992; **12**: 480–4.

A66 Jenyo MS, Asekun-Olarinmoye EO. Prevalence of scoliosis in secondary school children in Osogbo, Osun State, Nigeria. *Afr J Med Med Sci* 2005; **34**: 361–4.

A67 Keskin D, Bodur H, Acar F, Sureyya Boyacigil, Keskin G, Yucel M. School screening for scoliosis in Turkish children*. Eur J Phys Rehabil Med* 1997; **7**: 42–45.

A68 Koukourakis I, Giaourakis G, Kouvidis G, Kivernitakis E, Blazos J, Koukourakis M. Screening school children for scoliosis on the island of Crete. *J Spinal Disord* 1997; **10**: 527–31.

A69 Laulund T, Sojbjerg JO, Horlyck E. Moire topography in school screening for structural scoliosis**.** *Acta Orthop Scand* 1982; **53**: 765–8.

A70 Liston C. Evaluation of school screening for scoliosis in Western Australia. *Aust J Physiother* 1981; **27**: 37–43.

A71 Lonstein JE. Screening for spinal deformities in Minnesota schools. *Clin Orthop Relat Res* 1977; **126**: 33–42.

A72 Lonstein JE, Bjorklund S, Wanninger MH, Nelson RP. Voluntary school screening for scoliosis in Minnesota. *J Bone Joint Surg Am* 1982; **64**: 481–8.

A73 Luk KD, Lee CF, Cheung KM et al. Clinical effectiveness of school screening for adolescent idiopathic scoliosis: a large population-based retrospective cohort study. *Spine* 2010; **35**: 1607–14.

A74 Mittal RL, Aggerwal R, Sarwal AK. School screening for scoliosis in India. The evaluation of a scoliometer. *Int Ortho* 1987; **11**: 335–8.

A75 Morais T, Bernier M, Turcotte F. Age- and sex-specific prevalence of scoliosis and the value of school screening programs. *Am J Public Health* 1985; **75**: 1377–80.

A76 Newman DC, DeWald RL. School screening for scoliosis. *IMJ Ill Med J* 1977; **151**: 31–4.

A77 Nussinovitch M, Finkelstein Y, Amir J, Greenbaum E, Volovitz B. Adolescent screening for orthopedic problems in high school. *Public Health* 2002; **116**: 30–2.

A78 O'Brien JP, Van Akkerveeken PF. School screening for scoliosis: results of a pilot study. *Practitioner* 1977; **219**: 739–42.

A79 Oh KS, Chuah SL, Harwant S. The need for scoliosis screening in Malaysia. *Med J Malaysia* 2001; **56** Suppl C: 26–30.

A80 Ohtsuka Y, Yamagata M, Arai S, Kitahara H, Minami S. School screening for scoliosis by the Chiba University Medical School screening program. Results of 1.24 million students over an 8-year period. *Spine* 1988; **13**: 1251–7.

A81 Pin LH, Mo LY, Lin L, Hua LK, Hui HP, Hui DS, et al. Early diagnosis of scoliosis based on school-screening. *J Bone Joint Surg Am* 1985; **67**: 1202–5.

A82 Pruijs JE, van der Meer R, Hageman MA, Keessen W, van Wieringen JC. The benefits of school screening for scoliosis in the central part of The Netherlands. *Eur Spine J* 1996; **5**: 374–9.

A83 Randall FM, Denton TE. Scoliosis screening: a school survey. *Ala J Med Sci* 1983; **20**: 395–6.

A84 Robitaille Y, Villavicencio-Pereda C, Gurr J. Adolescent idiopathic scoliosis: epidemiology and treatment outcome in a large cohort of children six years after screening. *Int J Epidemiol* 1984; **13**: 319–23.

A85 Rogala EJ, Drummond DS, Gurr J. Scoliosis: incidence and natural history. A prospective epidemiological study. *J Bone Joint Surg Am* 1978; **60**: 173–6.

A86 Roubal PJ, Freeman DC, Placzek JD. Costs and effectiveness of scoliosis screening. *Physiotherapy* 1999; **85**: 259–268.

A87 Smyrnis PN, Valavanis J, Alexopoulos A, Siderakis G, Giannestras NJ. School screening for scoliosis in Athens. *J Bone Joint Surg Br* 1979; **61-B**: 215–7.

A88 Soucacos PN, Soucacos PK, Zacharis KC, Beris AE, Xenakis TA. School-screening for scoliosis. A prospective epidemiological study in northwestern and central Greece. *J Bone Joint Surg Am* 1997; **79**: 1498–503.

A89 Stirling AJ, Howel D, Millner PA, Sadiq S, Sharples D, Dickson RA. Late-onset idiopathic scoliosis in children six to fourteen years old. A cross-sectional prevalence study. *J Bone Joint Surg Am* 1996; **78**: 1330–6.

A90 Thilagaratnam S. School-based screening for scoliosis: is it cost-effective? *Singapore Med J* 2007; **48**: 1012–7.

A91 Velezis MJ, Sturm PF, Cobey J. Scoliosis screening revisited: findings from the District of Columbia. *J Pediatr Orthop* 2002; **22**: 788–91.

A92 Willner S, Uden A. A prospective prevalence study of scoliosis in Southern Sweden. *Acta Orthop Scand* 1982; **53**: 233–7.

A93 Willner S. Development of trunk asymmetries and structural scoliosis in pre-pubertal school children in Malmo: follow-up study of children 10-14 years of age. *J Pediatr Orthop* 1984; **4**: 452–5.

A94 Wong HK, Hui JH, Rajan U, Chia HP. Idiopathic scoliosis in Singapore schoolchildren: a prevalence study 15 years into the screening program. *Spine* 2005; **30**: 1188–96.

A95 Wynne EJ. Scoliosis: to screen or not to screen. *Can J Public Health* 1984; **75**: 277–80.

A96 Yawn BP, Yawn RA, Hodge D et al. A population-based study of school scoliosis screening. *JAMA* 1999; **282**: 1427–32.

A97 Yawn BP, Yawn RA. The estimated cost of school scoliosis screening. *Spine* 2000; **25**: 2387–91.

A98 Zhang GP, Li ZR, Wei XR, Cao YL, Cui QL. Screening for scoliosis among school children in Beijing. *Chin Med J (Engl)* 1988; **101**: 151–4.

**Program Effectiveness:**

A99 Bunge EM, Juttmann RE, de Koning HJ, Steering Committee of the NESCIO Group. Screening for scoliosis: do we have indications for effectiveness? *J Med Screen* 2006; **13**: 29–33.

A100 Bunge EM, Juttmann RE, van Biezen FC et al. Estimating the effectiveness of screening for scoliosis: a case-control study. *Pediatrics* 2008; **121**: 9–14.

A101 Goldberg CJ. (1995). Idem A59.

A102 Lonstein JE. (1982). Idem A72.

A103 Montgomery F, Willner S. Screening for idiopathic scoliosis. Comparison of 90 cases shows less surgery by early diagnosis. *Acta Orthop Scand*.1993; **64**: 456–8.

A104 Ohtsuka Y. (1998). Idem A80.

A105 Prujis JE. (1996). Idem A82.

A106 Soucacos PN. (1997). Idem A88.

A107 Torell G, Nordwall A, Nachemson A. The changing pattern of scoliosis treatment due to effective screening. *J Bone Joint Surg Am* 1981; **63**: 337–41.

A108 Wiergersma PA, Hofman A, Zielhuis GA. The effect of school screening on surgery for adolescent idiopathic scoliosis. *Eur J Public Health* 1998; **8**: 237–240.

A109 Willner S. (1982). Idem A92.

**Cost effectiveness:**

A110 Grivas T B, Vasiliadis E, Maziotou C, Savvidou D O. The direct cost of “thriasio” school screening program. *Scoliosis* 2007; **2**: 1–6.

A111 Koukourakis I, Giaourakis G, Kouvidis G, Kivernitakis E, Blazos J, Koukourakis M. Screening school children for scoliosis on the island of Crete. *J Spinal Disord* 1997; **10**: 527–31.

A112 Lee CF, Fong DY, Cheung KM, Cheng JC, Ng BK, Lam TP, et al. Costs of school scoliosis screening: a large, population-based study. *Spine* 2010; **35**: 2266–72.

A113 Morais T. (1985). Idem A75.

A114 Roubal PJ. (1999). Idem A86.

A115 Thilagaratham S. (2007). Idem A90.

A116 Ugras AA, Yilmaz M, Sungur I, Kaya I, Koyuncu Y, Cetinus ME. Prevalence of scoliosis and cost-effectiveness of screening in schools in Turkey. *J Back Musculoskelet Rehabil* 2010; **23**: 45–8.

A117 Yawn BP. (2000). Idem A97.

**Brace Effectiveness:**

A118 Allington NJ, Bowen JR. Adolescent idiopathic scoliosis: treatment with the Wilmington brace. A comparison of full-time and part-time use. *J Bone Joint Surg Am* 1996; **78**: 1056–62.

A119 Cottalorda J, Kohler R, Garin C, Genevois P, Lecante C, Berge B. Orthoses for mild scoliosis: a prospective study comparing traditional plaster mold manufacturing with fast, noncontact, 3-dimensional acquisition. *Spine* 2005; **30**: 399–405.

A120 Danielsson AJ, Nachemson AL. Radiologic findings and curve progression 22 years after treatment for adolescent idiopathic scoliosis: comparison of brace and surgical treatment with matching control group of straight individuals. *Spine* 2001; **26**: 516–25.

A121 Danielsson AJ, Hasserius R, Ohlin A, Nachemson AL. A prospective study of brace treatment versus observation alone in adolescent idiopathic scoliosis: a follow-up mean of 16 years after maturity. *Spine* 2007; **32**: 2198–207.

A122 den Boer WA, Anderson PG, v Limbeek J, Kooijman MA. Treatment of idiopathic scoliosis with side-shift therapy: an initial comparison with a brace treatment historical cohort. *Eur Spine J* 1999; **8**: 406–10.

A123 el-Sayyad M, Conine TA. Effect of exercise, bracing and electrical surface stimulation on idiopathic scoliosis: a preliminary study. *Int J Rehabil Res* 1994; **17**: 70–4.

A124 Fernandez-Feliberti R, Flynn J, Ramirez N, Trautmann M, Alegria M. Effectiveness of TLSO bracing in the conservative treatment of idiopathic scoliosis. *J Pediatr Orthop* 1995; **15**: 176–81.

A125 Gammon SR, Mehlman CT, Chan W, Heifetz J, Durrett G, Wall EJ. A comparison of thoracolumbosacral orthoses and SpineCor treatment of adolescent idiopathic scoliosis patients using the Scoliosis Research Society standardized criteria. *J Pediatr Orthop* 2010; **30**: 531–8.

A126 Ganjavian MS, Behtash H, Ameri E, Khakinahad M. Results of Milwaukee and Boston braces with or without metal marker around pads in patients with idiopathic scoliosis. *Acta Med Iran* 2011; **49**: 598–605.

A127 Gepstein R, Leitner Y, Zohar E et al. Effectiveness of the Charleston bending brace in the treatment of single-curve idiopathic scoliosis. *J Pediatr Orthop 2002*; **22**: 84–7.

A128 Howard A, Wright JG, Hedden D. A comparative study of TLSO, Charleston, and Milwaukee braces for idiopathic scoliosis. *Spine* 1998; **23**: 2404–11.

A129 Janicki JA, Poe-Kochert C, Armstrong DG, Thompson GH. A comparison of the thoracolumbosacral orthoses and providence orthosis in the treatment of adolescent idiopathic scoliosis: results using the new SRS inclusion and assessment criteria for bracing studies. *J Pediatr Orthop* 2007; **27**: 369–74.

A130 Katz DE, Richards BS, Browne RH, Herring JA. A comparison between the Boston brace and the Charleston bending brace in adolescent idiopathic scoliosis. *Spine* 1997; **22**: 1302–12.

A131 Katz DE, Herring JA, Browne RH, Kelly DM, Birch JG. Brace wear control of curve progression In adolescent idiopathic scoliosis*. J Bone Joint Surg Am* 2010; **92**: 1343–52.

A132 Labelle H, Bellefleur C, Joncas J, Aubin CE, Cheriet F. Preliminary evaluation of a computer-assisted tool for the design and adjustment of braces in idiopathic scoliosis: a prospective and randomized study. *Spine* 2007; **32**: 835–43.

A133 Nachemson AL, Peterson LE. Effectiveness of treatment with a brace in girls who have adolescent idiopathic scoliosis. A prospective, controlled study based on data from the Brace Study of the Scoliosis Research Society. *J Bone Joint Surg Am* 1995; **77**: 815–22.

A134 Negrini S, Marchini G. Efficacy of the symmetric, patient-oriented, rigid, three-dimensional, active (SPoRT) concept of bracing for scoliosis: a prospective study of the Sforzesco versus Lyon brace. *Eura Medicophys* 2007; **43**: 171-81; discussion 183–4.

A135 Weiss HR, Weiss GM. Brace treatment during pubertal growth spurt in girls with idiopathic scoliosis (IS): a prospective trial comparing two different concepts. *Pediatr Rehabil* 2005; **8**: 199–206.

A136 Wong MS, Lee JT, Luk KD, Chan LC. Effect of different casting methods on adolescent idiopathic scoliosis. *Prosthet Orthot Int* 2003; **27**: 121–31.

A137 Wong MS, Cheng JC, Lo KH. A comparison of treatment effectiveness between the CAD/CAM method and the manual method for managing adolescent idiopathic scoliosis. *Prosthet Orthot Int* 2005; **29**: 105–11.

A138 Wong MS, Cheng JC, Wong MW, So SF. A work study of the CAD/CAM method and conventional manual method in the fabrication of spinal orthoses for patients with adolescent idiopathic scoliosis. *Prosthet Orthot Int* 2005; **29**: 93–104.

A139 Wong MS, Cheng CY, Ng BK, Lam TP, Chiu SW. A comparison of the clinical effectiveness of spinal orthoses manufactured using the conventional manual method and CAD/CAM method in the management of AIS. *Stud Health Technol Inform* 2006; **123**: 225–32.

A140 Wong MS, Cheng JC, Lam TP et al. The effect of rigid versus flexible spinal orthosis on the clinical efficacy and acceptance of the patients with adolescent idiopathic scoliosis*. Spine* 2008; **33**: 1360–5.

A141 Yrjonen T, Ylikoski M, Schlenzka D, Kinnunen R, Poussa M. Effectiveness of the Providence nighttime bracing in adolescent idiopathic scoliosis: a comparative study of 36 female patients. *Eur Spine J* 2006; **15**: 1139–43
